# Yoga as an Intervention for the Reduction of Symptoms of Anxiety and Depression in Children and Adolescents: A Systematic Review

**DOI:** 10.3389/fped.2020.00078

**Published:** 2020-03-13

**Authors:** Aurora James-Palmer, Ellen Z. Anderson, Lori Zucker, Yana Kofman, Jean-Francois Daneault

**Affiliations:** ^1^Department of Rehabilitation and Movement Sciences, Rutgers University, Newark, NJ, United States; ^2^The Yoga Way Therapy Center, Morristown, NJ, United States

**Keywords:** child, adolescent, anxiety disorder, depressive disorder, mental health, complementary therapies, exercise, yoga

## Abstract

**Purpose:** The purpose of this review is to evaluate the implementation and effectiveness of yoga for the reduction of symptoms of anxiety and depression in youth. To our knowledge, there are no systematic reviews to date looking at the reduction of symptoms of both anxiety and depression.

**Methods:** Numerous scientific databases were searched up to November 2018 for experimental studies assessing changes in symptoms of anxiety and/or depression in youths following yoga interventions. Quality and level of evidence were assessed, and information was synthesized across studies.

**Results:** Twenty-seven studies involving youth with varying health statuses were reviewed. Intervention characteristics varied greatly across studies revealing multiple factors that may impact intervention efficacy, however 70% of the studies overall showed improvements. For studies assessing anxiety and depression, 58% showed reductions in both symptoms, while 25% showed reductions in anxiety only. Additionally, 70% of studies assessing anxiety alone showed improvements and 40% of studies only assessing depression showed improvements.

**Conclusion:** The studies reviewed, while of weak to moderate methodological quality, showed that yoga, defined by the practice of postures, generally leads to some reductions in anxiety and depression in youth regardless of health status and intervention characteristics.

Mental health conditions in youth are an increasing public health concern (1). According to the Center of Disease Control and Prevention (CDC), one in six children aged 2–8 years-old is diagnosed with a mental or behavioral disorder (2) and according to a national survey of 13–18 years-old, ~1 in every 4–5 adolescents has a mental disorder with severe impairment during their lifetime (3). This can negatively impact academic performance (4, 5), and future life outcomes (6, 7). The four most common mental health conditions in youth are attention-deficit hyperactivity disorder (ADHD), anxiety, conduct disorder, and depression (2). These can be divided into externalizing conditions (ADHD and conduct disorder) which often result in disruptive behaviors, and internalizing conditions (anxiety and depression) which present with psychological and physiological symptoms (8). While they may not be as easily observable, internalizing conditions lead to disruptions in academic performance and impaired function, and are highly prevalent (5, 9).

In the United States, as of 2016, there were 4.4 and 1.9 million children/adolescents diagnosed with anxiety and depression, respectively (2). Moreover, symptoms of anxiety and depression can be present in youths that do not have a clinical diagnosis. Therefore, a significantly larger number of youth than the number captured by the prevalence rates above must cope with symptoms of anxiety and depression in their daily lives. Anxiety is characterized by excessive fear and worry manifesting in disproportionate worrisome thoughts, increased heart rate, muscle tension, along with other somatic and behavioral disturbances (10). Youth with depression also exhibit somatic and behavioral deficits, and these are accompanied by excessive sadness and an empty or irritable mood (11). Both symptoms of anxiety and depression negatively impact school performance (5, 9). For instance, youth with anxiety withdraw from school prematurely compared to their peers; stating that they feel too nervous in school, feel uncomfortable leaving home, or feel intimidated by teachers and peers (5). Depression, on the other hand, manifests in poor academic performance secondary to school phobia and developmental behavior problems (9). Additionally, youth with anxiety (6, 7, 12) or depression (6) are more likely to have substance use disorder, and/or anxiety and depressive disorder in adulthood. Standard treatments for youth with anxiety and/or depression include cognitive behavioral therapy (CBT) (13, 14) and pharmaceutical interventions using selective serotonin reuptake inhibitors (SSRIs) (15, 16) or tricyclic antidepressants (17). However, a national survey showed that 80% of youth 6–17 years old who were defined as needing mental healthcare did not receive it (18). Parents' perceived barriers to mental healthcare include cost, cultural barriers, stigma, transportation, and access to mental healthcare providers (19). Minimizing these barriers and/or offering other interventions to help manage mental health concerns is warranted (16).

One such intervention is yoga. A multifaceted ancient practice, the purpose of yoga is to promote well-being through the integration of mind and body with an emphasis on self-realization (20). Within the tradition of yoga there are six branches that facilitate this path to well-being. Each branch is unique in its focus: (1) *Raja* (meditation and contemplation), (2) *Bhakti* (devotion), (3) *Jnana* (knowledge), (4) *Karma* (service), (5) *Tantra* (ritual), and (6) *Hatha* (physical postures). For example, an individual who practices *Karma Yoga* may devote their life to the service of others like Mother Teresa or someone who practices *Jnana Yoga* may study ancient yogic texts. The most well-known and commonly practiced element of yoga in the western world is physical postures. For the purpose of this review, yoga will refer to the practice of postures. It is important to note that this practice is a small piece of the yoga tradition and is informed through the values and philosophy of the other branches. Interestingly, there has been a significant increase in yoga practiced by youths aged 4–17 years-old. The 2017 National Health Interview Survey revealed that ~4.9 million youth reported practicing yoga in the United States, an increase of 5.3% since 2012 (21). Yoga, as a holistic tradition anchored in practice of physical poses, has been proposed to offer both mental and physical benefits (22–25). Schools, in particular, have increased the implementation of yoga in educational curriculums in response to increased stressors as a means to positively impact overall student health including fitness, mental health, social relationships, and self-awareness (23). Healthcare institutions have also begun to implement yoga to manage disease-sequela including pain, anxiety, depression, fatigue, and insomnia (22). Research exploring the potential underlying mechanisms of anxiety and depression reduction following yoga interventions is in development and primarily focuses on adult populations. However, there is preliminary evidence supporting the notion that physiological mechanisms such as improved regulation of the autonomic nervous system and increased thalamic GABA levels may help to explain the effect of yoga on anxiety and depression (26–28).

The use of yoga for the treatment of anxiety and depression, among other childhood mental health conditions, led to the publication of several research articles on this topic. However, it is challenging for clinicians and other stakeholders to discern how yoga should be implemented to achieve the best mental health outcomes. The limited research specific to yoga and pediatric mental health conditions has generally focused on externalizing conditions due to the more heavily pronounced behavioral disruptions (8). For example, two recent systematic reviews focused on the impact of yoga and/or meditation on youth with ADHD (29, 30). To our knowledge, only one review examined the specific impact of yoga on internalizing conditions in youth, which focused on anxiety (25). This previous review only examined studies that assessed yoga's impact on symptoms of anxiety in youth, while this current review examines studies assessing the impact of yoga on both symptoms of anxiety and depression. Additionally, since the earlier review's publication in 2015 there have been several new studies published that are in need of review and synthesis. The current review is intended to synthesize the available literature for both anxiety and depression in one document; providing a focused, yet comprehensive, resource of information for clinicians.

## Methods

This systematic review was conducted in accordance with PRISMA Guidelines (31) and the Cochrane Handbook for Systematic Reviews of Interventions (32).

### Literature Search

The following databases were searched by the first author for applicable articles published up to November 2018: PubMed, Scopus, Web of Science, PEDro, CINAHL, PsycINFO, and the Cochrane Library. Reference lists of relevant articles retrieved from database searches were hand searched for additional applicable articles. A variety of search terms, developed by a research librarian and the first author, utilized in different combinations with the use of the Boolean operators “AND” and “OR” as well as “^*^,” were as follows: infant, child, adolescent, newborn, baby, toddler, teen, boy, girl, youth, pediatric, school, preschool, yoga, *Iyengar, Ashtanga, Hatha, asana*, anxiety, anxiety disorder, panic, depression, depressive disorder, attention deficit disorder with hyperactivity, and ADHD. Search strategies were modified for each database, and MeSH terms were used when applicable. All authors reviewed and approved the search terms and strategies used to obtain the articles screened and reviewed, however the search was not physically done in duplicate by multiple authors.

### Inclusion Criteria

Experimental studies published in English including randomized control trials (RCT), controlled trials, cohort studies, case control trials, and case series were eligible for inclusion in this review. Studies were considered eligible if they included youth ≤18 years-old. If studies included participants >18 years-old but had a mean age range plus standard deviation of ≤18 years-old the studies were eligible for inclusion. Diagnosis of anxiety or depression was not a basis for inclusion or exclusion. Instead, studies were considered eligible if they included standardized outcome measures or subscales from a composite measure to assess symptoms of anxiety and/or depression. Studies were considered eligible if they implemented yoga, defined by the use of postures, as an intervention. Studies may have included the additional elements of breathing, meditation, and relaxation, but if studies included only breathing and/or meditation practices they were not included in this review.

### Data Management

#### Study Eligibility Screening and Full-Article Assessment

The inclusion and exclusion criteria outlined above were used to guide the eligibility screening and full-article assessment. Rayyan QCRI (33) was used to screen titles and abstracts of articles with at least 15% of the titles and abstracts screened by two authors for reliability. Following this screening, potential articles were further reviewed for inclusion with a full-text article assessment in which at least 20% of the articles were reviewed by two authors. Conflicts were resolved by an additional blinded reviewer. In total, there were 28 conflicts during the abstract screen and four conflicts during the full-article assessment.

#### Data Extraction From Included Articles

Data extracted from each article included: authors and year published, country where the study was conducted, study type, comparison conditions, number of subjects including age and sex, description of population, yogic elements, supplementary interventions, frequency and duration of yoga intervention, setting, delivery method, outcome measures, and effectiveness of the intervention. Over 15% of the data extraction was completed by two authors initially, however, the first author re-checked data extraction for all included articles.

#### Quality Appraisal

The Quality Assessment Tool for Quantitative Studies Effective Public Health Practice Project (34) was used to assess the level of bias of each study. Each article was also assigned a level of evidence according to the Original Centre for Evidence-Based Medicine (OCEBM) Levels of Evidence within the category of “Treatment Benefits” (35). Each article was appraised independently by two different authors. Due to varied interpretations of scoring criteria, there were nine conflicts overall, all of which were resolved by a third appraiser.

## Results

**Figure 1:**
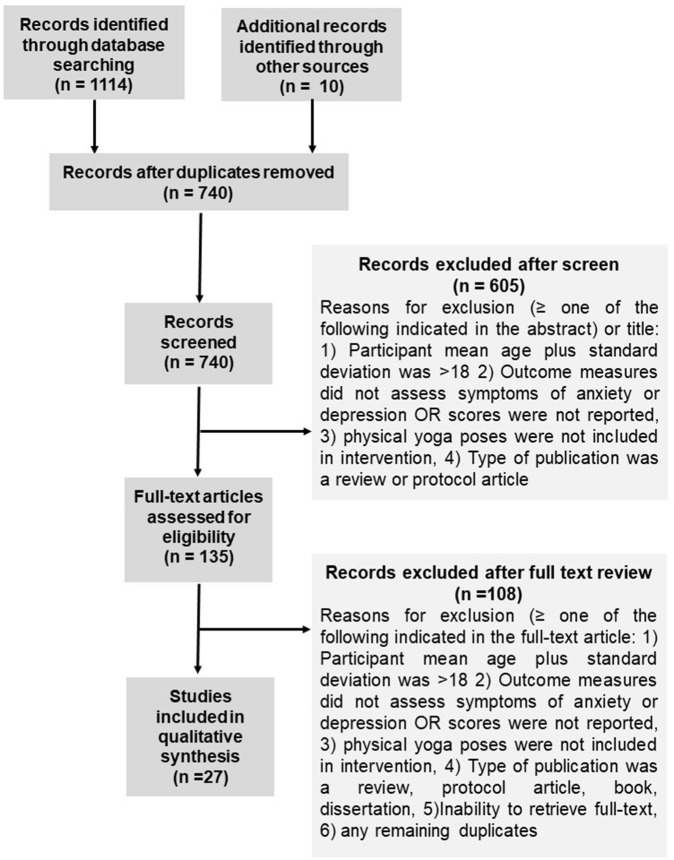
PRISMA study flow diagram illustrating results of database searches, results of eligibility assessments, and results of final inclusion.

The database search resulted in 1,114 references, with a total of 740 once duplicates were removed. Following the title/abstract screen, 135 references remained. Out of those, 27 were retained for final qualitative analysis. See Figure 1 for the PRISMA flow diagram (31). Selected articles were published between 2006 and 2018. Twenty-one of the 27 studies were conducted in the United States (36–55), two were conducted in India (56, 57), and one each in Germany (58), Canada (59), Iran (60), and Colombia (61). See Tables 1–3 for details regarding studies assessing both symptoms of anxiety and depression (Table 1), symptoms of anxiety (Table 2), and symptoms of depression (Table 3).

**Table 1:** Details for included studies assessing symptoms of anxiety and depression.

**References & Country**	**Study design & quality**	**Population**	**Yoga intervention**	**Comparison intervention**	**Outcomes & results**
	***1. Study type*** ***2. Quality of evidence*** ***3. Level of evidence***	***1. # of subjects: enrolled (N), in yoga group (Ny), & in comparison group (Nc)*** ***2. % of males*** ***3. Age range, mean (SD)*** ***4. Description***	***1. Yogic elements (in order as described)*** ***2. Frequency & duration*** ***3. Additional interventions*** ***4. Setting (group OR individual)***		***1. Anxiety*** ***2. Depression*** ***3. Adherence/study completion rate*** ***4. Adverse events***
Freedenberg et al. (53) USA	1. Randomized control trial 2. Moderate 3. Level 2	1. N = 46, Ny = 26, Nc = 20 2. 33% males 3. 12–18 years-old, 14.8 (1.7) 4. Adolescents with a cardiac diagnosis	1. Breathing, postures, meditation, & imagery 2. 1.5 h x1/week for 6 weeks 3. Included group discussions 4. NR (NR)	Video online discussion group, 1 hfor 6 weeks	1. HADS: NSD B/G or W/G 2. HADS: NSD B/G or W/G 3. 95% completed the study 4. NR
Velasquez et al. (61) Colombia	1. Randomized control trial 2. Weak 3. Level 3	1. N = 125, Ny = 68, Nc = 57 2. NR 3. NR 4. 5th, 8th, & 9th graders	1. Postures, breathing, relaxation, & meditation 2. 2 h × 24 times 3. No additional interventions 4. School (NR)	No treatment	1. Strength & difficulties questionnaire: ↓^*^ 2. Strength & difficulties questionnaire: ↓^*^ (*baseline as covariant negates this)* 3. NR 4. NR
Khalsa et al. (39) USA	1. Randomized control trial 2. Moderate 3. Level 2	1. N = 74, Ny = 74, Nc = 74 (group breakdown is unclear) 2. NR 3. 15–19 year-olds, 16.8 (0.6) 4. 11th & 12th graders at rural secondary school	1. Breathing, postures, & visualization 2. 30 min −40 min, 2–3 xs/week, 23–32 sessions for 11 weeks 3. Also included games 4. School (group)	Regular physical activity	1. BASC-2: NSD B/G or W/G POMS: NSD B/G or W/G 2. BASC-2: NSD B/G or W/G POMS: NSD B/G or W/G 3. Attendance to yoga classes comparable to attendance to PE classes 4. NR
Noggle et al. (62) USA	1. Randomized control trial 2. Weak 3. Level 3	1. N = 51, Ny = 36, Nc = 15 2. 51% male in yoga group 3. Mean age 17 years-old 4. 11th, 12th graders	1. Postures & relaxation 2. 2–3 sessions/week for 10 weeks (28 sessions total) 3. No additional interventions 4. School (group)	PE control, 30–40 min, 2–3 times/week, 10 weeks	1. POMS: ↓^*^ 2. POMS: ↓ 3. Moderate attendance noted 4. 1 adverse event reported due to unknown pre-existing condition
Kuttner et al. (59) Canada	1. Randomized control trial 2. Moderate 3. Level 2	1. N = 28, Ny = 14, Nc = 14 2. 29% males 3. 11–18 years-old, 14.15 (1.95) 4. Children with irritable bowel syndrome	1. Postures & breathing 2. 10 min/day for 4 weeks 3. No additional interventions 4. 1 in-person, others at home with DVD (individual)	Subjects were asked to manage IBS as typical	1. RCMAS: ↓ 2. CDI-short form: NSD 3. 79% completed the study 4. NR
Sams et al. (45) USA	1. Cohort^+^ 2. Weak 3. Level 3	1. N = 65, Ny = 65, Nc = 0 2. Unclear 3. 13–17 years-old, 15.06 (1.34) 4. various mental health diagnoses	1. Breathing, postures, & mindfulness 2. A single 50 min session 3. Mindful activities 4. Psychiatric inpatient unit (group)	None	1. POMS: ↓^**^ W/G 2. POMS: ↓^**^ W/G 3. NR 4. NR
Tejvani et al. (57) India	1. Cohort^+^ 2. Weak 3. Level 3	1. N = 34, Ny = 34, Nc = 0 2. males 79% males 3. 12–20 years old, 12.47 4. Residents of an orphanage	1. Postures, breathing, deep relaxation, & meditation 2. 1 h/day for 6 days/week for 2 weeks 3. No additional interventions 4. Orphanage (NR)	None	1. HADS: ↓^**^ W/G 2. HADS: ↓^**^ W/G 3. NR 4. NR
Hall et al. (54) USA	1. Cohort^+^ 2. Weak 3. Level 3	1. N = 29, Ny = 20, Nc = 0 2. 0% males 3. 14–18 years-old, 15.9 (1.8) 4. Adolescents with DSM-5 diagnosis of an eating disorder	1. Breathing, chanting, & postures 2. 60–90 min yoga/week for 12 sessions 3. No additional interventions 4. Local studio (NR)	None	1. STAI: ↓^*^ (*state only)* W/G 2. SOM: ↓^**^ W/G 3. 69% completed study 4. NR
McNamara et al. (40) USA	1. Cohort^+^ 2. Weak 3. Level 3	1. N = 21, Ny = 20, Nc = 0 2. Males 38% males 3. 7–20 years-old, 11 (NR) 4. Children with cystic fibrosis	1. Postures 2. 40 min, 6xs over 10 weeks 3. Counseling 4. Hospital clinic (individual)	None	1. STAIC: ↓^*^ W/G immediately pre & post single session HADS: NSD W/G 2. HADS: NSD W/G 3. 95% completed the study 4. NR
Freedenberg at al. (52) USA	1. Cohort^+^ 2. Weak 3. Level 3	1. N = 10, Ny = 10, Nc = 0 2. 60% males 3. 12–18 years-old, 15 (1.94) 4. Adolescents with implantable cardioverter defibrillators or pacemakers	1. Breathing, postures, meditation, & imagery 2. 1.5–2 h x1/week for 6 weeks 3. Included group discussions 4. At cardiology clinic (group)	None	1. HADS: ↓^*^ W/G 2. HADS: NSD W/G 3. 60% attended all yoga sessions 4. 1 subject was noted as depressed at end of the intervention (causation not determined)
Frank et al. (51) USA	1. Cohort^+^ 2. Weak 3. Level 3	1. N = 49, Ny = 49, Nc = 0 2. 45% males 3. Age NR 4. 9–12th graders in an alternative school	1. Breathing, postures, & meditation 2. 30 min x3–4 3. Taught coping skills, building awareness, understanding stress 4. School (group)	None	1. BSI: ↓^*^ W/G 2. BSI: ↓^*^ W/G 3. NR 4. NR
Benavides and Caballero (36) USA	1. Cohort^+^ 2. Weak 3. Level 4	1. N = 20, Ny = 20, Nc = 0 2. NR 3. 8–15 years-old, 11.7 (1.5) 4. Children at risk for type 2 diabetes	1. Postures, breathing, & meditation 2. 1.25 h x3/week for 12 weeks 3. No additional interventions 4. NR (NR)	None	1. BAI-Y: *subjects ↑baseline levels* ↓W/G 2. BDI-Y: *subjects ↑baseline levels* ↓W/G 3. 70% completed the study 4. NR

*Summary of study characteristics for studies assessing symptoms of anxiety and depression. Study characteristics summarized include country in which study was conducted, study type, quality of evidence, level of evidence, number of subjects in the study and in each group, population age range and mean age, a description of the population, yogic elements included in the intervention, frequency and duration of the intervention, additional intervention components included, setting, if the intervention was a group intervention or individual, a description of comparison interventions, and pertinent study results. The studies in this table are organized first by study type starting with randomized control trials, followed by cohort analytic studies, and then cohort studies. Within each section of specific study type, studies are organized by year published, from the most recent study to most dated study. NR, Not reported; PE, physical education; NSD, no significant difference or change; W/G, within group; B/G, between group, STAI, State Trait-Anxiety Inventory; RCMAS, Revised Children's Manifest Anxiety Scale; BAI-Y, Beck Anxiety Inventory for Youth; BDI-Y, Beck Depression Inventory for Youth; CDI, Children's Depression Inventory; BRUMS, Brunel University Mood Scale; BSI, Brief Symptoms Inventory; HADS, Hospital Anxiety and Depression Scale; BASC-2, Behavior Assessment System for Children; POMS, Profile of Mood States; SDQ, Strength & Difficulties Questionnaire; ↓, decrease; ↑, increase*;

* *p ≥ 0.05*,

** *p ≥ 0.001*;

+ *, only within group differences could be examined due to study design*.

**Table 2:** Details for included studies assessing symptoms of anxiety.

**References & Country**	**Study design & quality**	**Population**	**Yoga intervention**	**Comparison intervention**	**Outcomes Used & Results**
	***1. Study type*** ***2. Quality of evidence*** ***3. Level of evidence***	***1. No. of subjects: enrolled (N), in yoga group (Ny), & in comparison group (Nc)*** ***2. % of males*** ***3. Age range, mean (SD)*** ***4. Description***	***1. Yogic Elements (in order as described)*** ***2. Frequency & duration*** ***3. Additional interventions*** ***4. Setting (group OR individual)***		***1. Anxiety*** ***2. Depression*** ***3. Adherence/study completion rate*** ***4. Adverse events***
Moody et al. (42) USA	1. Randomized control trial 2. Moderate 3. Level 3	1. N = 70, Ny = 25, Nc = 35 2. 41% males 3. 5–21 years-old 4. Children hospitalized diagnosed with sickle cell disease vaso-occlusive crisis	1. Mindfulness, postures, & breathing, relaxation 2. 30 min, avg 2.5 sessions completed 3. No additional interventions 4. Hospital (individual)	30 min with music and yoga instructor present	1. STAIC: ↓ w/G for both groups, NSD B/G 2. N/A 3. NR 4. 2 adverse events occurred per group (investigators deemed intervention was not the cause)
Quach, et al. (43) USA	1. Randomized control trial 2. Weak 3. Level 3	1. N = 198, Ny = 65, Nc = wait-list control (N = 57) & mindfulness meditation (N = 61) 2. 38% males 3. 12–15 years old, 13.18 (.72) 4. 11th graders	1. Breathing & postures 2. 45 min x2/week for 4 weeks 3. Discussion 4. School (groups of 10–13)	2 comparison groups- (1) wait-list control (2) mindfulness meditation with breathing and discussion	1. SCARED: NSD B/G, NSD W/G 2. N/A 3. 87% completed the study 4. NR
Nidhi et al. (56) India	1. Randomized control trial 2. Moderate 3. Level 2	1. N = 72, Ny = 25, Nc = 35 2. 0% males 3. 15–18 years-olds 4. Girls with a diagnosis of polycystic ovarian syndrome	1. Postures, breathing, relaxation, & meditation 2. 60 min x7/week for 12 weeks 3. 1 h counseling 4. Residential school (group)	Physical exercise, non-yogic breathing, supine rest, individual counseling	1. STAIC: ↓^*^ (trait) B/G, NSD (state) B/G Statistical significance W/G not reported 2. N/A 3. 75% completed the study 4. NR
Khalsa et al. (38) USA	1. Cohort analytic 2. Weak 3. Level 3	1. N = 135, Ny = 84, Nc = 51 2. 44% males 3. Mean- 16 years-old 4. Residential music students of a prestigious summer program	1. Breathing, postures, & relaxation, meditation 2. 60 min x3/week for 6 weeks 3. No additional interventions 4. Music summer camp (group)	No treatment	1. STAI: 2007 sample ↓^*^ (trait only) B/G, 2008 sample-NSD, 2007+2008- NSD W/G, NSD B/G, statistical significance W/G N/R 2. N/A 3. Average of 17 yoga sessions attended, yoga group 89% completed thy study, 86% for control group 4. NR
Conn et al. (48) USA	1. Cohort^+^ 2. Weak 3. Level 4	1. N = 40, Ny = 40, Nc = 0 2. 83% males 3. 6–12 years-old, 9.45 (NR) 4. Children who have survived burn injuries (severity of burns = 5–75%)	1. Postures, breathing, meditation, & visualization 2. 1 h session x4 3. Yoga games, coping strategies, and meaningful messages 4. Summer camp for children with burn injuries (group by age)	None	1. YEQ scale: ↓^**^ W/G 2. N/A 3. Participants were included in the study (final analysis) only if they complete all 4 yoga sessions 4. NR
Richter et al. (58) Germany	1. Cohort 2. Weak 3. Level 3	1. N = 25, Ny = 12, Nc = 12 2. 48% males 3. 6–11 years-old, 8.4 (1.4) 4. School children	1. Postures 2. 45 min 2x/week for 6 weeks 3. Children's story given with postures 4. School over holidays (NR)	Physical skills training group, free-play, and movement tasks	1. BAV 3-11: NSD W/B & B/G 2. N/A 3. 96% completed the study 4. NR
Hooke at al. (55) USA	1. Cohort^+^ 2. Weak 3. Level 3	1. N = 18, Ny = 13, Nc = 0 2. 17% males 3. 10–17 years-old 4. Children and adolescents who had been treated for cancer	1. Seated meditation, breathing, postures, & a final resting pose 2. 45 min x1/week for 6 weeks 3. No additional interventions 4. Research institutions (group)	None	1. STAIC: Children ↓^*^ W/G Adolescents ↓ W/G 2. N/A 3. 62% completed the study 4. NR
Steiner, et al. (46) USA	1. Cohort^+^ 2. Weak 3. Level 3	1. N = 41, Ny = 37, Nc = 0 2. 59% males 3. 8–11 years-old, 10.4 (0.82) 4. 4th & 5th graders, various mental health diagnoses	1. Relaxation, postures, & meditation 2. 1 hour 2x/week for 14 weeks 3. Imagery, social component 4. School (groups of 7–10)	None	1. STAI-C: ↑^*^ (state) W/G, ↑ (trait) W/G 2. N/A 3. 90% completed the study 4. NR
Moemeni et al. (60) Iran	1. Cohort+ 2. Weak 3. Level 3	1. N = 135, Ny = 135, Nc = 0 2. 50% males 3. 6 year-olds 4. Preschoolers with anxiety	1. Postures, breathing, & meditation 2. 30 min x3/week for 12 weeks 3. No additional interventions 4. School (NR)	None	1. RCMAS: ↓^*^ W/G 2. N/A 3. NR 4. NR
Thygeson et al. (47) USA	1. Cohort+ 2. Weak 3. Level 3	1. N = 16, Ny = 16, Nc = 0 2. 63% males 3. 6–12 years-old (*N* = 11)13-18 years-old (*N* = 5)4. Children hospitalized with a diagnosis of cancer	1. Meditation, postures, & relaxation 2. Single 45-min session 3. No additional interventions 4. Children's Hospital (group)	None	1. STAI: adolescents ↓^*^ W/G, children NSD W/G 2. N/A 3. 94% completed the study 4. NR

*Summary of study characteristics for studies assessing symptoms of anxiety. Study characteristics summarized include country in which study was conducted, study type, quality of evidence, level of evidence, number of subjects in the study and in each group, population age range and mean age, a description of the population, yogic elements included in the intervention, frequency and duration of the intervention, additional intervention components included, setting, if the intervention was a group intervention or individual, a description of comparison interventions, and pertinent study results. The studies in this table are organized first by study type starting with randomized control trials, followed by cohort analytic studies, and then cohort studies. Within each section of specific study type, studies are organized by year published, from the most recent study to most dated study. NR, Not reported; PE, physical education; NSD, no significant difference or change; W/G, within group; B/G, between group; STAI, State Trait-Anxiety Inventory; RCMAS, Revised Children's Manifest Anxiety Scale; BAI-Y, Beck Anxiety Inventory for Youth; BAV-11, Bochumer angstverfahren fur Kinder im Vor- und Grundschulalter; YEQ, Yoga Evaluation Questionnaire; SCARED, Child Related Anxiety Disorders; BSI, Brief Symptoms Inventory; HADS, Hospital Anxiety and Depression Scale; BASC-2, Behavior Assessment System for Children; POMS, Profile of Mood States; ↓, decrease; ↑, increase*;

* *p ≥ 0.05*,

** *p ≥ 0.001*;

+ *, only within group differences could be examined due to study design*.

**Table 3:** Details for included studies assessing symptoms of depression.

**References & Country**	**Study design & quality**	**Population**	**Yoga intervention**	**Comparison intervention**	**Outcome measure & results**
	***1. Study type*** ***2. Quality of evidence*** ***3. Level of evidence***	***1. # of subjects: enrolled (N), in yoga group (Ny), & in comparison group (Nc)*** ***2. % of males*** ***3. Age range, mean (SD)*** ***4. Description***	***1. Yogic elements (in order as described)*** ***2. Frequency & duration*** ***3. Additional interventions*** ***4. Setting (group OR individual)***		***1. Anxiety*** ***2. Depression*** ***3. Adherence/study completion rate*** ***4. Adverse events***
Butzer et al. (37) USA	1. Randomized control trial 2. Weak 3. Level 2	1. N = 211, Ny = 117, Nc = 94 2. 37% males 3. Mean age 12.64 (0.33) 4. 7th grade students	1. Breathing, postures, & relaxation 2. 45 min 1–2x/ week for 32 sessions 3. No additional interventions 4. School (group)	Regular PE class	1. N/A 2. BRUMS: both groups ↑^*^ W/G, NSD B/G 3. 94% from yoga group completed the study & 96% from control group 4. NR
Fishbein et al. (50) USA	1. Randomized control trial 2. Weak 3. Level 3	1. N = 104, Ny = 45, Nc = 40 2. 46% males 3. 14–20 year-olds, 16.7 (Ns) 4. 9–12th graders with risk for dropping out	1. Meditation, general stretching, postures, & meditation 2. 50 min x3/ week for 7 weeks (20 sessions) 3. Principles/mindfulness themes 4. School (Ns)	Regularly scheduled class	1. N/A 2. BRUMS: NSD W/G, NSD B/G 3. 82% completed the study 4. NR
Mendelson et al. (41) USA	1. Cohort analytic 2. Weak 3. Level 2	1. N = 97, Ny = 51, Nc = 46 2. 15% males 3. mean age 10.15 4. 4th and 5th graders at risk for stress and other mental health conditions	1. Postures, breathing, & mindfulness 2. 45 min 4x/week for 12 weeks 3. No additional interventions 4. School (25 per group)	Regularly scheduled class	1. N/A 2. SMFQ-C: NSD W/G, NSD B/G 3. 5 dropouts 95% completed 4. NR
Felver et al. (49) USA	1. Cohort^+^ 2. Weak 3. Level 3	1. N = 58, Ny = 58, Nc = 58 (all subjects in both groups) 2. Not specified 3. mean age = 15.75 (SD = 9 months) 4. 9th & 10th graders	1. Breathing, postures, & relaxation 2. 35 min x5/week for 3 non-consecutive weeks (data was collected pre-post one session) 3. yoga alternated with regular PE, data was collected pre/post 1 of each class 4. School (group)	Regularly scheduled PE class- capture the flag, (data collected 1 week after yoga data collected)	1. N/A 2. BRUMS: ↓^*^ W/G & B/G 3. 81% completed the study 4. NR
Rosenblatt et al. (44) USA	1. Cohort^+^ 2. Weak 3. Level 3	1. N = 33, Ny = 24, Nc = 0 2. 67% males 3. 3–16 years-old, 8.9 (3.6) 4. Children with a diagnosis of Autism-spectrum disorder	1. Breathing, postures, & relaxation 2. 45 min x8 (weeks NR) 3. Music and dance 4. Hospital (group)	None	1. N/A 2. BASC-2: ↓^*^ W/G for latency age group, NSD W/G with all ages pooled 3. 76% completed the study 4. NR

*Summary of study characteristics for studies assessing symptoms of depression. Study characteristics summarized include country in which study was conducted, study type, quality of evidence, level of evidence, number of subjects in the study and in each group, population age range and mean age, a description of the population, yogic elements included in the intervention, frequency and duration of the intervention, additional intervention components included, setting, if the intervention was a group intervention or individual, a description of comparison interventions, and pertinent study results. The studies in this table are organized first by study type starting with randomized control trials, followed by cohort analytic studies, and then cohort studies. Within each section of specific study type, studies are organized by year published, from the most recent study to most dated study. NR, Not reported; PE, physical education; NSD, no significant difference or change; W/G, within group; B/G, between group; BDI-Y, Beck Depression Inventory for Youth; CDI, Children's Depression Inventory; SMFQ-C, Short Mood and Feelings Questionnaire child version; BDI, Beck Depression Inventory; BRUMS, Brunel University Mood Scale; BSI, Brief Symptoms Inventory; HADS, Hospital Anxiety and Depression Scale; BASC-2, Behavior Assessment System for Children; POMS, Profile of Mood States; ↓, decrease; ↑, increase*;

* *p ≥ 0.05*;

** *p ≥ 0.001*;

+ *= only within group differences could be examined due to study design*.

### Types and Quality of Studies

Ten studies were RCT (37, 39, 42, 43, 50, 52, 56, 59, 61, 62), 15 were cohort studies (36, 40, 44–49, 51, 52, 54, 55, 57, 58, 60), and two were cohort analytic (38, 41). Control and comparison group activities varied significantly across studies. Yoga was compare to no treatment groups (38, 61), regularly scheduled physical education (PE) classes (37, 49, 62), non-yogic physical activity (39, 56), regular classroom activities (41, 50), physical skills training (58), matched time with music (42) and usual care for symptoms of irritable bowel syndrome (IBS) (59). Additionally, one RCT compared yoga to mindfulness in a wait-list control design (43). The methodological quality of the studies ranged from weak to moderate, with five studies of moderate quality (39, 42, 53, 56, 59), and 22 studies of low quality (36–38, 40, 41, 43–48, 50–52, 54, 57, 58, 60–62). The OCEBM level of evidence ranged from two to four. Six studies were level two (37, 39, 41, 53, 56, 59), 19 were level three (38, 40, 42–47, 49–52, 54, 55, 57, 58, 60–62), and two were level four (36, 48).

### Participants

The age of participants ranged from 3 to 21 years-old. One study included individuals up to 21 years-old (42), while three studies included individuals up to 20 years-old (40, 50, 57). The 22 studies that reported number of participants of each sex showed an average of 41% male participants (37, 38, 40–44, 46–48, 50–60, 62). The total number of subjects included in each study ranged from 10 to 211. Specific diagnoses/descriptions included a risk factor for type 2 diabetes (36), cardiac diagnosis (53) or devices (52), burn injuries (48), cancer (47, 55), eating disorders (54), abnormal levels of anxiety (60), IBS (59), cystic fibrosis (40), sickle cell disease vaso-occlusive crisis (42), polycystic ovarian syndrome (56), Autism-spectrum disorder (ASD) (44), individuals in an in-patient psychiatric unit (45), residents of an orphanage (57), and students at an elite music summer program (38). The remaining studies conducted involved schoolchildren from 4th to 12th grade within school settings (37, 39, 41, 43, 46, 49–51, 58, 61, 62).

### Yoga Interventions

#### Yogic Elements

The type of yoga implemented was not clearly reported or defined across studies. Therefore, this information was not included in the review. Information regarding the use of yogic elements, however, was more clearly presented and was thus included, as follows. As part of the inclusion criteria, all studies utilized yoga and thus included the yogic element of physical postures. Breathing, meditation, and relaxation were also commonly utilized. The definitions of mindfulness, meditation, and relaxation varied between studies. During data extraction the elements of mindfulness and meditation were grouped together, but relaxation was extracted separately due to the nature of the descriptions across studies. Eight studies utilized postures, breathing, and meditation (36, 41, 45, 48, 51–53, 60), while three utilized postures, breathing, and relaxation (37, 44, 49). Six studies used all four yogic elements of postures, breathing, meditation, and relaxation (38, 42, 55–57, 61). Two studies used postures, meditation, and relaxation (46, 47), four studies utilized postures and breathing (39, 43, 54, 59), one study used postures and relaxation (62), and one used postures and meditation (50). Only two studies used physical postures alone (40, 58). In addition to the above elements, some studies included other intervention components including imagery, visualization, chanting, yoga games, stretching, dance therapy, discussions, and philosophy (Tables 1–4). Ten studies specified using a pre-designed yoga curriculum (37–39, 45, 46, 49, 51–53, 62).

**Table 4:**
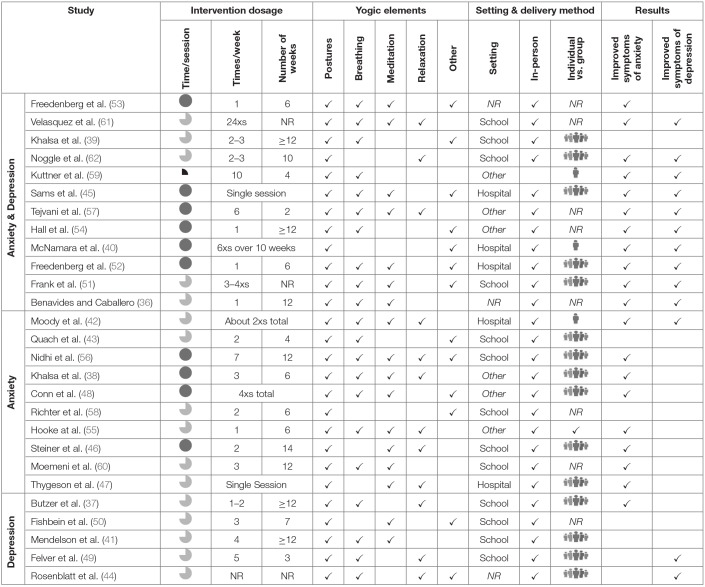
Intervention details.

*Summary of intervention details, by study, including frequency and duration, yogic elements (postures, breathing, meditation, relaxation, and other), setting (school, healthcare institution, or other setting), delivery method (if it was an individual or group intervention and if it was delivered in-person or delivered via other means), and improvements noted. Studies are organized to coincide with Tables 1–3. NR, Not reported, hospital indicates any healthcare institution like a clinic, psychiatric institution, or hospital. 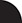 ≤ 30 min, 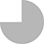 = 30 to <60 min, 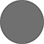 = ≥ 60 min, ✓ = yes, 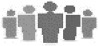 = group intervention, 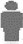 = individual intervention*.

#### Delivery Method and Setting

Three studies implemented the intervention individually (40, 42, 59), 16 were conducted in groups (37–39, 43–49, 51, 52, 55, 56, 62) and eight studies did not specify how the intervention was implemented (36, 50, 53, 54, 57, 58, 60, 61). Groups were assigned by sex, age, gym class, or randomly assigned. All the studies, except one, occurred via in-person instruction. This study included an in-person instructional session, but subsequent intervention sessions occurred at home through a DVD yoga program (59). Thirteen studies were conducted in a school setting (37, 39, 41, 43, 46, 49–51, 56, 58, 60–62) one was in a local yoga studio (54) and another took place in an orphanage (57). Seven studies occurred at a healthcare institution (40, 42, 44, 45, 47, 52, 55), including two in-patient settings (42, 45). Two studies were conducted at summer camps, one being for adolescent musicians (38) and the other for youth surviving burn injuries (48). Lastly, two studies did not specify the intervention setting (36, 53) (Table 4).

#### Duration and Frequency

Intervention duration and frequency was heterogenous across studies, ranging from a single session to repetitive sessions spread over 14 weeks. Length of intervention ranged from 10 min to 2 h, and frequency ranged from one time per week to daily (Table 4).

### Outcome Measures

Each study included at least one outcome measure used to assess symptoms of anxiety and/or depression. Twelve studies utilized and reported results from outcome measures to assess both anxiety and depression (36, 39, 40, 45, 51–54, 57, 59, 61, 62), 10 studies assessed anxiety (38, 42, 43, 46–48, 55, 56, 58, 60), and five studies assessed depression (37, 41, 44, 49, 50). See Tables 1–3 for specific details on the outcome measures used.

Outcome measures varied across studies and were primarily self-report questionnaires with some parent and teacher reports. The most common measure used to assess anxiety was the State Trait-Anxiety Inventory (STAI) (38, 40, 42, 46, 47, 54, 56). Other measures included the Revised Children's Manifest Anxiety Scale (RCMAS) (59, 60), Beck Anxiety Inventory for Youth (BAI-Y) (36), Bochumer angstverfahren fur Kinder im Vor- und Grundschulalter (BAV-11) (58), Yoga Evaluation Questionnaire (YEQ) (48), and Screen for Child Related Anxiety Disorders (SCARED) (43). Anxiety subscales from composite outcome measures included the Brief Symptoms Inventory (BSI) (51), Hospital Anxiety and Depression Scale (HADS) (52, 53, 57), Behavior Assessment System for Children (BASC-2) (39), Strength and Difficulties Questionnaire (61), and Profile of Mood States (POMS) (39, 45, 62).

Depression-specific measures were the Beck Depression Inventory for Youth (BDI-Y) (36), Children's Depression Inventory (CDI) (59), Short Mood and Feelings Questionnaire child version (SMFQ-C) (41), and the Beck Depression Inventory (BDI) (54). Depression subscales of larger composite outcome measures included the Brunel University Mood Scale (BRUMS) (37, 49, 50), HADS (52, 53, 57), POMS (39, 45, 62), BSI (51), BASC-2 (39, 44), and Strength and Difficulties Questionnaire (61).

### Effectiveness of the Yoga Intervention by Condition

#### Studies Assessing Anxiety and Depression

There were five RCTs assessing changes in levels of both anxiety and depression (39, 53, 59, 61, 62). Of those, two showed no significant differences between or within groups (39, 53) while two studies showed reductions in symptoms of both anxiety and depression (61, 62), and one study only showed reductions in symptoms of anxiety (59). The two studies showing improvements across both outcomes compared yoga to no treatment (61) or regular PE class (62), and the study showing improvements in anxiety compared yoga to usual care for the treatment of IBS (59). Studies showing no differences between groups compared yoga to a discussion group (53) and regular physical activity, but the frequency and duration of the regular physical activity was not specified (62). Four out of seven studies assessing yoga in a pre-post uncontrolled design showed improvements for both outcomes following the intervention (45, 51, 54, 57). Another study (36) only showed improvements for subjects with elevated baseline levels of anxiety and depression, and two studies showed reductions in symptoms of anxiety only (40, 52) (Table 1).

#### Studies Assessing Only Anxiety

When comparing yoga to another intervention, three out of five studies showed some improvements in anxiety (38, 42, 56). A study comparing yoga to physical exercise showed decreased trait anxiety in the yoga group, but no changes in state anxiety (56). Another study showed a decrease in trait anxiety in the yoga group for one out of two of their yearly samples, but no change when the samples were pooled (38). A study comparing yoga to a time matched control in which participants listened to music showed a decrease in anxiety levels within both groups but no significant difference between groups (42). A RCT comparing breathing and physical yoga postures to breathing and meditation, and waitlist controls found no significant differences in anxiety levels within or between groups (43). A study comparing yoga to a physical skills training group showed no significant differences within or between groups (58). Four of the five studies examining the effectiveness of yoga at managing anxiety using a single group design showed a decrease in the severity of anxiety (40, 47, 48, 55, 60), while one showed an increase in anxiety (46) (Table 2).

#### Studies Assessing Only Depression

One out of five studies assessing depression showed significant changes within and between groups on the depression subscale of the BRUMS outcome measure when comparing a yoga session to a regularly scheduled PE class (49). This study was unique in that subjects participated in both conditions alternating across three non-consecutive weeks, but data was only collected pre and post one yoga session and one PE class; which could have influenced results. Three other studies assessing depression found no significant difference within or between groups when comparing yoga to regularly scheduled PE or academic classes (37, 41, 50). Lastly, one study assessing depression for youth with ASD analyzed the results for all ages and for subjects yet to have full manifestation of ASD finding significant reductions in depression on the BASC-2 for the subset of the subjects yet to have full manifestation of ASD, but not for all subjects when pooled (44) (Table 3).

#### Study Completion and Adherence

Study completion rate was reported in 16 studies (84%) (36, 38, 40, 41, 43, 44, 46, 47, 49, 50, 53–56, 58, 59). Adherence to yoga classes was rarely reported, but one study reported 100% adherence to the yoga training (52), while another reported 94% adherence (37). One study characterized adherence as comparable to attendance to typical PE classes (39) and another characterized it as moderate (62).

#### Adverse Events

Adverse events were explicitly reported in three studies (42, 52, 62). One study reported that one subject was depressed following the intervention, but causation was not specified (52). Another study, conducted in an acute care setting, reported two adverse events occurring in the yoga group and three in the control group. Events occurring in the yoga group included a vascular necrosis and acute splenic sequestration, and events in the control group were two cases of acute chest syndrome and one occurrence of suicidal ideation. It was reported that the adverse events were unlikely caused by the study (42). Lastly, one study determined that the single adverse event was due to an undisclosed pre-existing condition (62). In this case the adverse event was Valsalva retinopathy resulting in transient blindness in one eye following an inverted posture. Vision returned without any medical intervention and the participant discontinued the yoga classes.

## Discussion

The purpose of this review was to evaluate the implementation and effectiveness of yoga for children and adolescents with symptoms of anxiety and depression. We identified 12 studies assessing symptoms of anxiety and depression, 10 assessing symptoms of anxiety, and five assessing symptoms of depression. A majority assessed these symptoms as secondary outcomes within another primary objective. Overall, 70% of the studies showed some type of improvement in symptoms of anxiety and/or depression. For studies assessing anxiety and depression, 58% showed improvement in both symptoms, while 25% showed improvements in anxiety only. For studies only assessing anxiety, 70% showed improvements and 40% of studies only assessing depression showed improvements. Overall methodological quality of evidence was weak which can be attributed to lack of randomization, blinding, and limited analysis. Unfortunately, this finding is similar to other reviews indicating that little improvements have been made in methodological quality of yoga studies following previous recommendations (22, 24, 25).

Due to many factors that may have impacted efficacy, it is challenging to break apart each study to determine why improvements may have or may not have occurred. Thus, this discussion will focus on factors that may have impacted efficacy. For more detailed accounts regarding each study, see Tables 1–4.

### Participants

The age, gender, and health status of participants varied across settings. Most studies were conducted with youth exhibiting specific conditions including IBS, cystic fibrosis, cancer, and burn injuries or with healthy schoolchildren. This revealed that research specifically regarding youth with clinical diagnoses of anxiety and/or depression is rare, with only two studies examining this issue (45, 46). Nonetheless, the available literature supports the use of yoga to address symptoms of anxiety and depression in varying pediatric populations.

### Yogic Elements

Due to limited reporting and unclear definitions, the type of yoga could not be extracted and synthesized in this review. However, the studies reported information about the yogic elements which were more clearly defined and described across studies. All studies included in this review implemented various forms of *yoga*, as defined through the use of postures, and a majority of the studies also included breathing, meditation, and/or relaxation. The definitions and descriptions of meditation, relaxation, and mindfulness overlapped throughout the studies. Studies typically indicated that an activity was considered meditation or mindfulness if there was a focus on awareness, whereas other studies mentioned the use of relaxation techniques without increased attention to body or mind awareness. For example, one study included a meditation exercise in which subjects were instructed to focus on their breath in *Savasana* and a relaxation technique in which participants were guided through a progressive relaxation exercise (42).

The use of physical postures as the primary yogic element occurred in two studies, both of which showed improvements in symptoms of anxiety (40, 58). All other studies utilized combinations of postures, breathing, meditation/mindfulness, and relaxation as the primary yogic elements. In addition to the primary yogic elements, supplementary interventions including visualization, chanting, dance, and discussion groups were utilized. Every combination of yogic elements listed in the results section demonstrated improvements in at least one study, except the one study that utilized only postures and meditation, which did not show any improvements. However, this is not to say that postures and meditation cannot be efficacious because the sample size was limited and there are many other factors that may have impacted the intervention's efficacy. Overall, physical yoga, regardless of whether other elements were included, appeared to result in improvements in symptoms of anxiety and/or depression.

### Delivery Method and Setting

The intervention settings varied across studies with no one setting emerging as best. All three studies with one-on-one interaction showed a decrease in symptoms of anxiety (40, 42, 59). However, due to the small sample, it is not possible to say conclusively whether this approach is more effective. Additionally, the one study involving at-home-yoga via DVD instruction (59), did show improvements in anxiety. Overall, this review shows that yoga classes delivered across a variety of settings including schools and healthcare institutions alike, generally result in reductions of symptoms of anxiety and depression, but more research is needed to explore individualized classes and alternate modes of delivery.

### Duration and Frequency

The frequency and duration of yoga interventions varied greatly across studies. Overall, studies not showing improvements (37–39, 41, 43, 46, 50, 53) involved interventions with frequencies four times per week or less and/or durations of 6 weeks or less. However, some studies that did show improvements in symptom severity involved interventions with frequencies and durations within these ranges; making it problematic to identify optimal dosage. This is most likely due to the many other factors contributing to the intervention effectiveness, including yogic elements, delivery method, and setting. While future research is needed to determine the optimal dosage, our results are in agreement with Weaver et al. who noted that interventions of higher frequencies and durations are typically associated with better outcomes (25).

### Feasibility and Safety

Sixteen studies (36, 38, 40, 41, 43, 44, 46, 47, 49, 50, 53–56, 58, 59) reported study completion rate which translated to ~84%. Adherence to yoga classes was only reported in four studies, with a range of moderate to high adherence (37, 39, 52, 62). This level of adherence indicates good feasibility however, limited reporting of adherence contributed to the low methodological quality ratings. Similarly, the reporting of adverse events was minimal across studies making it difficult to form conclusions about the safety of implementing yoga for youth with anxiety and depression. A previous systematic review regarding safety of yoga in adults supports this observation; concluding that while yoga appears as safe as usual exercise, adequate reporting of adverse events is crucial for future studies (63). Overall, this current review recommends more robust reporting of adherence and adverse events, including severity of event and relationship to the intervention.

### Outcome Measures

The outcome measures used in the studies may have impacted the results. They were standardized self-report and proxy-report measures that may not have been sensitive enough to evaluate a change in symptom severity. While the majority of measures used have adequate reliability and validity, not all of them have demonstrated validity and reliability in youth. For example, HADS and BRUMS have only been validated for adult populations. Some studies utilizing these outcomes demonstrated improvements while others did not. Additionally, floor effects, or low baseline values, may have also impacted the results. Some studies did not report baseline values and thus it was challenging to determine if a lack of improvement may have been due to minimal symptoms at baseline as opposed to poor efficacy of the intervention.

#### Relevant Previous Review

Weaver et al. in 2015 focused on the use of yoga to reduce anxiety in youth (25). The current review included 10 studies that were previously reported on by Weaver et al. (25, 36, 38, 39, 44, 46, 47, 51, 59, 60, 62), but also included an additional 17 studies [six of which assessed anxiety and depression (45, 52–54, 57, 61), seven assessed anxiety (40, 42, 43, 48, 55, 56, 58), and four assessed depression (37, 41, 49, 50)]. Overall, all but one study not included in the previous review were published after 2015. Weaver et al. (25) concluded that yoga could result in reductions of anxiety however, heterogenous methodology indicated a need for further research of higher quality. Unfortunately, only three studies published after 2015 were of moderate quality while 14 were of weak quality, indicating that study quality has not drastically improved since the previous review's publication.

#### Recommendations

Future yoga research should focus on improving methodological quality of studies through robust reporting and use of quality appraisal guides as checklist to gauge quality during the study design phase. Special attention should be given to clearly reporting details specified in these clinical appraisal tools including randomization, blinding, and statistical analysis. Along with improving methodological quality, reporting of the intervention characteristics should be more detailed. The yoga type should be clearly named and defined with a supporting rationale. The specific yoga elements included in the intervention should be named along with brief description of their implementation. Additionally, delivery method and setting should be clearly stated. Subject adherence to yoga sessions and reasons for absences should be reported. Adverse events, including a brief description, severity of the event, impact on the subject, and relationship to the study protocol should be reported. If adverse events do not occur, this should be explicitly stated. In addition to improving reporting, the research itself should begin to explore yoga in greater depth. Studies examining dosage and delivery methods should be conducted.

The reviewed available evidence does not lend itself to discrete recommendations due to the vast heterogeneity and low to moderate methodological quality. However, by integrating the clinical experience of the authors, the available evidence, and established principles and theories of yoga, physical therapy, and exercise, we recommend that future studies follow the below advice for intervention parameters when implementing yoga for the reduction of anxiety and depression in youth. Higher frequency and duration is typically associated with better outcomes, however, taking into account practicality and pediatric attention spans, an intervention should be implemented for at least 30 min, 2–3 times per week for ~6–12 weeks. There is not enough evidence to recommend a specific type of yoga. All the studies included in this review, however, utilized physical yoga postures, so future yoga interventions should employ physical postures and may include additional yogic elements such as breathing, meditation, and/or relaxation. Furthermore, reliable, valid, and age appropriate outcome measures should be used to assess symptoms of anxiety and depression in future studies. Finally, future researchers should seek to employ high methodological rigor to allow for optimal recommendations regarding the application of yoga with children who have symptoms of anxiety and depression.

#### Limitations

Initially, the purpose of this review was to investigate the implementation of yoga for children and adolescents with common mental health conditions. Outcome measures assessing ADHD and conduct disorder were considered for inclusion at the onset of this review. However, unlike those used for youth with symptoms of anxiety and depression, outcome measures used for youth with ADHD and conduct disorder varied greatly in what they measured (i.e., different aspects of behavior, cognitive constructs, etc.). Additionally, our preliminary background research revealed that a review of the pediatric yoga literature specific to internalizing symptoms had not been done. Yet the terms “attention deficit disorder” and “ADHD” were included in the database search terms. This evolution of our study purpose may have impacted the studies obtained during the database search. Secondly, the eligibility screening and data extraction components of the systematic review were not done in duplicate for all articles. Instead, over 15% of the articles were formally looked at by two reviewers. We checked for consistency between the two reviewers included in that 15% and did not increase the amount of overlap due to the nature and rare occurrence of the conflicts. Also, the first author re-checked all of the extracted data. Lastly, we limited our search to studies published in English.

## Conclusions

The purpose of this review was to evaluate the implementation and effectiveness of yoga for youth with the internalizing symptoms of anxiety and depression, providing a synthesized resource for clinicians. The studies reviewed, while of weak to moderate methodological quality, generally showed reductions in symptoms of anxiety and marginal reductions in symptoms of depression. While the variety of intervention characteristics made it challenging to recommend specific intervention parameters, it showed that yoga, defined here by the practice of postures, generally leads to some reductions in anxiety and depression regardless of type, other yoga elements practiced, delivery method, and setting. Future research will aid in the development of optimal dosage and intervention parameters. Currently, however, this review provides promising evidence supporting the use of yoga to address internalizing symptoms of mental health in pediatric populations.

## Author Contributions

AJ-P, EA, LZ, YK, and J-FD have made substantial contributions to the design of the work and to the acquisition, analysis, and interpretation of the data for the work, involved in either drafting the work or critically revising the work throughout its various stages, and provided final approval of this current version to be published and agree to be accountable for all aspects of the work ensuring that questions related to the accuracy or integrity of any part of the work are appropriately investigated and resolved.

### Conflict of Interest

The authors declare that the research was conducted in the absence of any commercial or financial relationships that could be construed as a potential conflict of interest.
